# Neurophysiological Approach by Self-Control of Your Stress-Related Autonomic Nervous System with Depression, Stress and Anxiety Patients

**DOI:** 10.3390/ijerph18073329

**Published:** 2021-03-24

**Authors:** Kees Blase, Eric Vermetten, Paul Lehrer, Richard Gevirtz

**Affiliations:** 1 National Centre Stress Management, Innovational and Educational Centre HartFocus, 1231 NC78 Loosdrecht, The Netherlands; 2 Department Psychiatry, Leiden University Medical Center, 2333 ZA Leiden, The Netherlands; h.g.j.m.vermetten@lumc.nl; 3 Rutgers Medical School, Rutgers University, Monmouth Junction, NJ 08852, USA; lehrer@rwjms.rutgers.edu; 4 California School of Professional Psychology, Alliant International University, San Diego, CA 92131, USA; rgevirtz@alliant.edu

**Keywords:** self-control, HRV, HRV-Biofeedback, PTSD, depression, anxiety, sleeping disorder, stress, psychophysiology, neurophysiology, Vagal Tone, coronary artery disease

## Abstract

Background: Heart Rate Variability Biofeedback (HRVB) is a treatment in which patients learn self-regulation of a physiological dysregulated vagal nerve function. While the therapeutic approach of HRVB is promising for a variety of disorders, it has not yet been regularly offered in a mental health treatment setting. Aim: To provide a systematic review about the efficacy of HRV-Biofeedback in treatment of anxiety, depression, and stress related disorders. Method: Systematic review in PubMed and Web of Science in 2020 with terms HRV, biofeedback, Post-Traumatic Stress Disorder (PTSD), depression, panic disorder, and anxiety disorder. Selection, critical appraisal, and description of the Random Controlled Trials (RCT) studies. Combined with recent meta-analyses. Results: The search resulted in a total of 881 studies. After critical appraisal, nine RCTs have been selected as well as two other relevant studies. The RCTs with control groups treatment as usual, muscle relaxation training and a “placebo“-biofeedback instrument revealed significant clinical efficacy and better results compared with control conditions, mostly significant. In the depression studies average reduction at the Beck Depression Inventory (BDI) scale was 64% (HRVB plus Treatment as Usual (TAU) versus 25% (control group with TAU) and 30% reduction (HRVB) at the PSQ scale versus 7% (control group with TAU). In the PTSD studies average reduction at the BDI-scale was 53% (HRV plus TAU) versus 24% (control group with TAU) and 22% (HRVB) versus 10% (TAU) with the PTSD Checklist (PCL). In other systematic reviews significant effects have been shown for HRV-Biofeedback in treatment of asthma, coronary artery disease, sleeping disorders, postpartum depression and stress and anxiety. Conclusion: This systematic review shows significant improvement of the non-invasive HRVB training in stress related disorders like PTSD, depression, and panic disorder, in particular when combined with cognitive behavioral therapy or different TAU. Effects were visible after four weeks of training, but clinical practice in a longer daily self-treatment of eight weeks is more promising. More research to integrate HRVB in treatment of stress related disorders in psychiatry is warranted, as well as research focused on the neurophysiological mechanisms.

## 1. Introduction

Heart Rate Variability (HRV) is a neurobiological marker of the autonomic nervous system (ANS) with decreased HRV indices being associated with a variety of negative physical and psychological outcomes [1,2,3,4,5]. Heart Rate Variability Biofeedback (HRVB) is a non-invasive treatment, in which patients are assumed to self-regulate a physiological dysregulated vagal nerve function by restoring the autonomic homeostasis [6,7,8]. This is relevant for stress-related disorders such as sleep disorders, anxiety, asthma, fibromyalgia, recovery of heart failure, and others. In this time of COVID-19 more knowledge about self-control, Digital Health and a balance in the Autonomic Nervous System (ANS) is relevant.

HRVB affects cardiovascular homeostatic reflexes by increasing flexibility and recovery from fight or flight adaptive situations [9]. Thousands of studies have been published about HRV and HRVB. Most of the studies are focused on HRV as marker for instance as predictor of physical outcomes anxiety disorders and PTSD, cancer recovery [10,11,12,13]. HRVB has been characterized as making visible the neurophysiological effect of meditation [14]. Work-related stress develops gradually and effects both the physical and mental health of those experiencing it, which can eventually lead to burnout. Work related stress symptoms include insomnia, sleep disturbances, menstrual disorders, irritation, and depression [2]. HRV represents the ability to adapt to stress and is a marker of physiological stress [1]. Higher HRV amplitude indicates better self-regulation and is associated with lower cardiovascular risk and alleviating symptoms of stress, anxiety [15,16,17]. A recent meta-analysis affirms the efficacy of HRVB with wearable devices on self-reported stress [18].

In this study different HRV-Biofeedback devices are reported like StressEraser, Infinity HRV-Biofeedback and Balance Manager. In this study we will focus on HRV-Biofeedback studies as additional treatment in clinical practice.

In 1996, the Taskforce of the European Society of Cardiology and the North American Society of Pacing and Electrophysiology formulated definitions of the various HRV metrics [1]. These international standards: High Frequency(HF), Very Low Frequency(VLF), and Low Frequency(LF) are derived by spectral analysis of the interbeat interval (RR).

VLF (very low frequency = 0.003–0.04 Hz), when measured over long time frames, has been interpreted as reflecting sympathetic activity (Action); LF (low frequency = 0.04–0.15 Hz) reflects the combination of sympathetic and vagal balance (Balance), though recent studies have questioned this interpretation, and HF (high frequency = 0.15–0.4 Hz) is interpreted as reflecting vagal activity (Calm/sleepy) (see Figure 2).

We can describe this as ABC: Action, Balance, or Calm/Sleepy, where A indicates dominance of VLF (Action in the mind or body)

**Figure 1:**
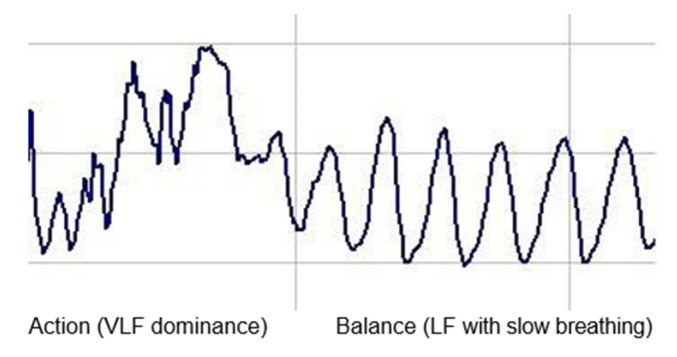
HRV (Heart Rate Variability) patterns (tachogram).

B indicates Balance between sympathetic and vagal activity or LF, and C indicates dominance of HF (Calm/sleepy) (see Figure 1).

**Figure 2:**
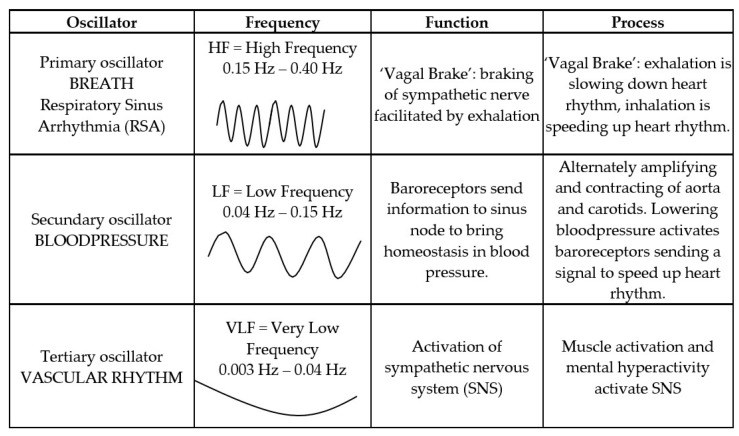
HRV as composition of 3 oscillation processes [7]

Autonomic Balance is the marker of the state of the ANS. Unconscious perception of safety is reflected in higher HF values, while threat produces HF (vagal) withdrawal and sympathetic activation. Autonomic balance is defined in terms of complex heart rate (HR) patterns that increase and decrease in response to respiratory fluctuations (Figure 2).

However, when clients shift to slow effortless breathing patterns (as shown above), they stimulate reflexes in the Autonomous Nervous System (ANS) and Central Nervous system (CNS) that, over time, “rewire” these systems so as to enhance ANS flexibility. This formula, during slow effortless breathing, LF: (VLF+LF+HF) can be used as an index of the client’s success in achieving their training goals. This formula is used in HRV-Biofeedback instruments Balance Manager and StressEraser Pro. After 20 years of research and clinical work, this formula was maybe more effective than LF:HF.

HRVB is a natural oscillation between the breathing cycle and heartrate. Inhalation temporarily suppresses vagal activity, causing a decrease in the inter-beat interval and an increase in heart rate; exhalation activates vagal activity, causing an increase in the inter-beat interval and a decrease in heart rate [19]. Heart rate is a dynamic function that varies in each moment. An HRV pattern (tachogram) is a composition of three oscillation processes: Respiratory Sinus Arrhythmia (RSA), baroreflex, and vascular rhythm [19].

Until 1996, HRV was primarily used as marker of the autonomic nervous system, but in 1996 Paul Lehrer (in collaboration with Evgeny Vaschillo) reported the observation that if you breath in the frequency of the baroreceptor and you slow down the tertiary oscillator (quietly not moving too much) than the ANS come into autonomic balance.

The body can be brought into the state of autonomic balance through guided breathing with HRV-Biofeedback. Breathing in the resonance frequency (between 0.05 and 0.15 Hz, which is the same as 4–7 breaths per minute) can be compared to guiding someone on a swing by pushing the swing at the correct moment to optimize their swing (resonance). Breathing at resonance frequency trains the reflexes of the cardiovascular system, in particular the baroreflex [19]. Breathing in the resonance frequency (resonance between respiratory and baroreflex rhythms) and using HRV-Biofeedback creates autonomic balance in the ANS and in this study we will show the effects of training Autonomic Balance with HRVB.

Long standing stress, PTSD and traumatic incidents can disturb stability of the Vagus nerve and create complex disturbances in heartbeat, HRV and hyperarousal, allowing overactivation in the Sympathetic Nervous System [6,20,21]. Our nervous system is continuously evaluating risk in the environment through an unconscious process of neuroception [22]. That is why it is innovative and important to integrate neurophysiological body-focused and self-regulating methods like HRVB in treatment of depression, trauma, and anxiety [23,24]. Reduced HRV amplitude has been found in patients with major depression disorder (MDD) [25]. HRV is a biological marker of the autonomic nervous system with decreased HRV indices being associated with MDD patients and probably being a biomarker of depression [26].

## 2. Method

**Figure 3:**
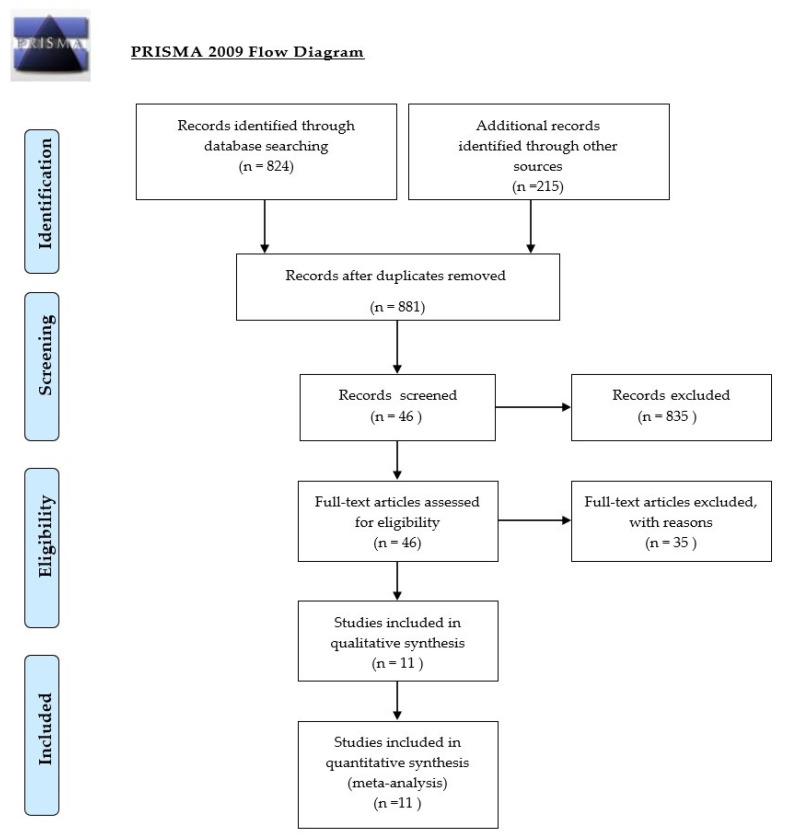
Flow diagram (for the selection of review articles).

In 2016, we published a systematic review and we now present an updated systematic review [7]. This review is based on searches in PubMed and Web of Science with an evidence- based critical review based on the GRADE method [27]. GRADE means Grades of Recommendation Assessment, Development and Evaluation. GRADE can be compared to the Prisma Statement with search terms: HRV (and synonyms) combined with PTSD, combat disorder, depression, depressed mood, anxiety, panic disorder (Figure 3).

Studies were screened by title and summary. This produced 881 articles. These articles have been judged by two raters with GRADE criteria, based on relevance (design, validation, setting, period, protocol, scale) and results (starting measures, result, significance, and missing data). The 46 selected articles have been screened again and the 11 selected studies are described in Table 1 in next chapter. Inclusion criteria were HRVB as clinical intervention of depression, PTSD and anxiety disorder with adults. Exclusion criteria: observational studies, anxiety studies with persons without disorders, children, other languages than English and German. Other meta-analyses of HRVB were added to find the evidence-based effects of HRVB treatment.

## 3. Results

Most studies were single blind studies except for studies of Karavidas and Siepman [35,39]. Double blind study is not possible in biofeedback studies. Al studies had a high validity in presenting starting description and quality of outcome data.

### 3.1. HRVB as Additional Treatment of Depression

One of the first single blind RCT HRVB studies was focused on 46 female depressed welfare-to-work recipients in California. The HRVB-group (*n* = 20) and the control group treated by progressive muscle relaxation (*n* = 26) received antidepressant medicine [30,40].

The HRVB group showed significant reduction in the Beck Depression Inventory (BDI) score from 35.0 (SD 8.0) towards 17, (SD 12.6) in the first 4 weeks and after 8 weeks even towards 7.8. In the control group, reduction was smaller: from 30.1 (SD 10.2) towards 16.9 (SD 13.3) in 8 weeks. Reduction of 78% HRVB, versus 44% in the control group.

Another early RCT study with StressEraser focused on 60 patients after myocard infarct [31]. HRVB was integrated with dialectic behavioral therapy in conjunction with sertraline medication. The control group only used sertraline medication. The reduction on BDI scale was 76% after 12 weeks daily exercising 20 min with HRVB (BDI from 30.9 towards 7.5) versus 29% reduction in control group (BDI from 30.5 towards 22.0).

A third RCT as a bio behavioral intervention for depressive symptoms in patients after cardiac surgery was in Italy [32]. HRVB group and control group, TAU group, received both TAU that consisted of daily counseling sessions such as dietary and smoking cessation counseling, weight management, and stress-management according to the guidelines of the American Heart Association and the American Association of Cardiovascular and Pulmonary Rehabilitation. The HRVB group added 2 weeks of daily 45 min biofeedback. The significant reduction measured by CES-D (Centre Epidemiological Study-Depression) scale after 2 weeks was 42% compared with the control group’s 1% reduction.

The psychophysiological mechanisms underlying depression as a risk factor for cardiovascular disease, cardiac morbidity, and fatal cardiac events after surgery are still debated. In the Netherlands, a scientific clinical consortium called Benefit is focusing on cardiac rehabilitation and prevention of cardiac diseases using lifestyle interventions and self-regulation programs such as non-smoking programs, personal lifestyle coaching, blended care with eHealth, HRV-Biofeedback, and mindfulness.

Another study in the Netherlands (University of Amsterdam), in cooperation with Paul Lehrer (USA), is a randomized controlled trial with 20 pregnant and 30 non-pregnant women, mean age 31.6 years [33,41]. The intervention consisted of a 5-week HRVB training with weekly 60–90 min sessions and daily exercises with StressEraser. Research has convincingly shown that high levels of maternal stress, anxiety, and depression during pregnancy are not only harmful for the women herself, but may also affect the child she is carrying. The control group was a wait-list group. The Depression Anxiety Stress Scale (DASS) and Pittsburg Sleep Quality Index (PSQI) were administered pre- and post-intervention. Effect sizes were larger in the HRVB group on all scales. The DASS depression scale women started with a score of 5.45 and after 5 weeks went down to 2.8 (reduction 49%). The wait-list reduction was 35%.

In total, two studies of the Kaohsiung Medical University in Taiwan were selected for review: HRVB with heroin users with depressive symptoms and patients with Major Depression Disorder [37,38]. The prevalence of major depressive episodes among heroin users has been found to be 25%. The nine participants had weekly sessions with HRVB software. They had a reduction of 20% on the BDI depression scale, and 25% on the BDI cognitive depression scale.

In 2019, at Kaohsiung Medical University, there was a RCT study with 48 participants with MDD depression and insomnia [38]. The HRVB group received weekly 60-min sessions for 6 weeks, and the control group received medical care only. The significant reduction at the BDI-II scale was 38 versus 1% in the control group. The significant reduction on the sleep disorder scale (PSQI) was 28% in the HRVB group and 5% in the control group. In 2019, at Alliant University in San Diego USA, a dissertation was presented with 37 participants with MDD and a Latino background [36]. There are different factors that appear to limit the access and quality of mental health care for Latinos, including lack of insurance, cultural barriers, stigma, distrust of medical providers, expression of distress, and fear of deportation. The HRVB participants received four HRV-Biofeedback sessions, were trained in diafragmatic breathing and finding their personal resonance frequency. They were provided with an app to be used as a pacer for daily exercising 10–20 min during 4 weeks in addition to sessions with their psychotherapist.

TAU participants completed four consecutive weeks of psychotherapy. Pre- and post-tests show 39.9% reduction in the Patient Health Questionnaire (PHQ-9): from 16.42 (SD2.93) towards 10.85 (SD6.81). With the TAU group reduction was 6.6% from 17.70 (SD 4.61) towards 16.52 (SD 6.57). In the Anxiety test (GAD-7) reduction was 41.3%: from 11.08 (SD3.40) towards 6.50 (SD3.37) versus TAU 8.8% reduction: from 15.36 (SD 4.41) towards 14.00 (SD4.56).

### 3.2. HRVB as Additional Treatment of PTSD

Although the connection between HRV and PTSD was already well-known the first RCT study of HRVB and PTSD was reported in 2009 [28,42,43,44]. In total, 76 participants were recruited from an urban residential therapeutic community program for the treatment of PTSD with comorbid substance use disorder. After randomization, 38 participants joined the 4 weeks daily program. The HRVB group trained 20 min daily with the StressEraser and the control group received daily 20 min Progressive Muscle Relaxation (PMR). The HRVB group had significantly (*p* = 0.001) greater reductions in depression scores compared to PMR (Progressive Muscle Relaxation) In HRVB group BDI-II reduced 53% (from 26.4 to 12.3) and 24% (from 25.95 to 19.47). On the scale, 29–63 = severe depression; 20–28 = moderate depression; 14–19 = mild depression; 0–13 = minimal depression. Reduction on PCL scale was 27% (HRVB) compared to 18% (PMR).

A second RCT study, with PTSD and HRVB, was focused on participants of US Department of Veteran Affairs—MEDVAMC [29]. In an 8 weeks program of weekly sessions with the Resonance Frequency protocol of Lehrer et al., veterans were trained in autonomic balance.

PCL score reduced significantly by 16% with HRVB while the TAU control group (only TAU) reduction after 8 weeks was 9% (not significant). Patients reported a satisfaction score of 8 (scale 1–10) and more than 50% wanted to continue using breathing in resonance frequency.

In 2014 a systematic review for psychiatric disorders with integration of treatment with HRVB was published [45]. In 2020, a systematic review and meta-analysis was published showing HRVB improves emotional and physical health, and performance [46]. Their initial review yielded 1868 papers from which 58 met inclusion criteria. HRVB has the largest effect sizes for depression (Hedge g = −0.72 and *p <* 0.0005), anger (g = −0.54 and *p <* 0.02), emotion regulation (g = −0.34 and *p <* 0.0005), asthma (g = −1.357), and athletic performance (g = −90), and smaller effect sizes on PTSD (g = 0.29) and quality of life (g = 0.14). The average effect size of the 58 studies for HRVB and paced breathing versus control conditions was found to be small to medium Hedge g = 0.37. High effect size g = −0.8 medium effect size: g = −0.5 and small effect size: g = −0.2.

In our study, we focalized on Depression, PTSD and Anxiety and with the GRADE method we got a smaller selection of 10 studies. Most of the selected studies showed a large effect size [46]:

[28] PTSD study: g = −0.739

[29] PTSD study: g = −0.296

[30] Depression: g = −0.748

[32] Depression: g = −0.958

## 4. Discussion

In the first review of RCT studies of Heart Rate Variability Biofeedback the conclusion was: ”a number of research studies have given at least tentative support for the effectiveness of HRVB for a wide range of medical and emotional disorders.”[19].

Below you can see the summary, with addition of the RCT studies after 2014 (Table 2):

While the first studies and RCTs were mostly done in USA, you can see the emergence of high quality studies from all over the world (Germany, the Netherlands, Belgium and the UK, South Korea, Taiwan, Japan, Brazil, and Australia).

In our manuscript the systematic review is focused on depression, PTSD, and anxiety. Effects were visible after 4 weeks of HRVB-training, but clinical practice in a longer daily self-treatment of 8 weeks showed more reduction on the BDI. Daily HRV-Biofeedback was more effective than weekly training, as we see in the studies of Rene and Chaudri with the StressEraser [31,40]. Perhaps three 8 min a day or two 10 min a day sessions are the most effective7.

Interestingly, in the systematic review it was stated: “an interesting implication of our findings is that length of treatment and home practice does not influence the effect size. Perhaps learning how to breathe at resonance frequency provides a sufficient method for most of the beneficial effects.”(p. 125) [46].

**Figure 4:**
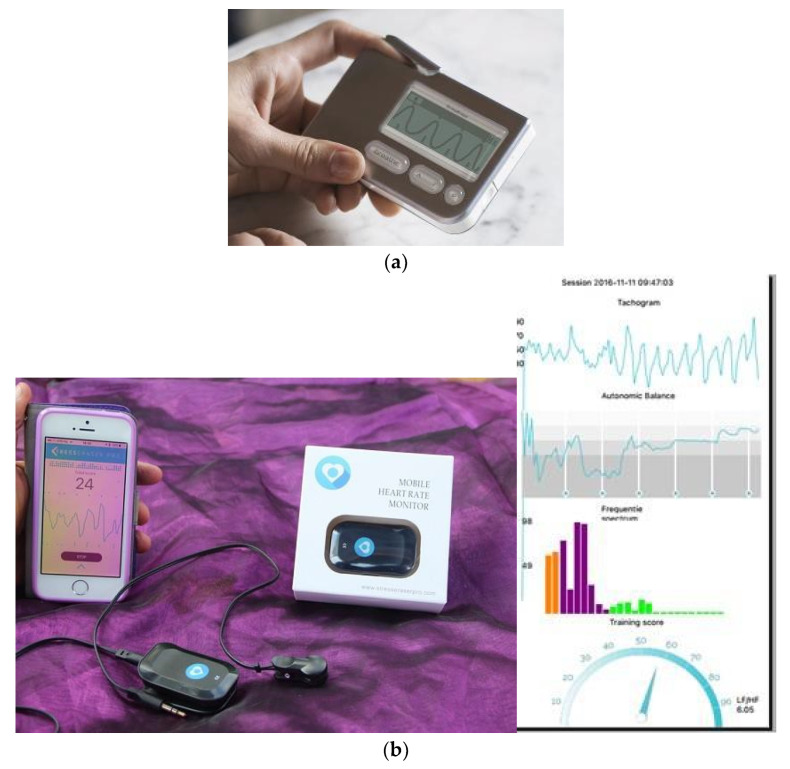
(**a**) StressEraser. (**b**) StressEraser Pro.

The StressEraser, used in many of the HRVB studies was a noninvasive portable handheld device attempting to increase RSA using a respiratory training system. A highly sensitive infrared light sensor detects tiny changes in the rate at which blood pulses through the fingertip. The finger sensor has a photoplethysmograph to identify every pulse. The StressEraser (Figure 4a) was a very effective device, because the resonance frequency (resonance between heart rhythm and breathing rhythm) was automatically seen on the screen. StressEraser is not any more available since 2015. However, the StressEraser Pro (Figure 4b) has been developed for iPhone with more detailed information of HRV patterns:

In this StressEraser Pro, you can read the tachogram, the frequency spectre with VLF (Orange), LF (purple), and HF (green) and the training effect score: LF/(VLF+ LF + HF).

**Figure 5:**
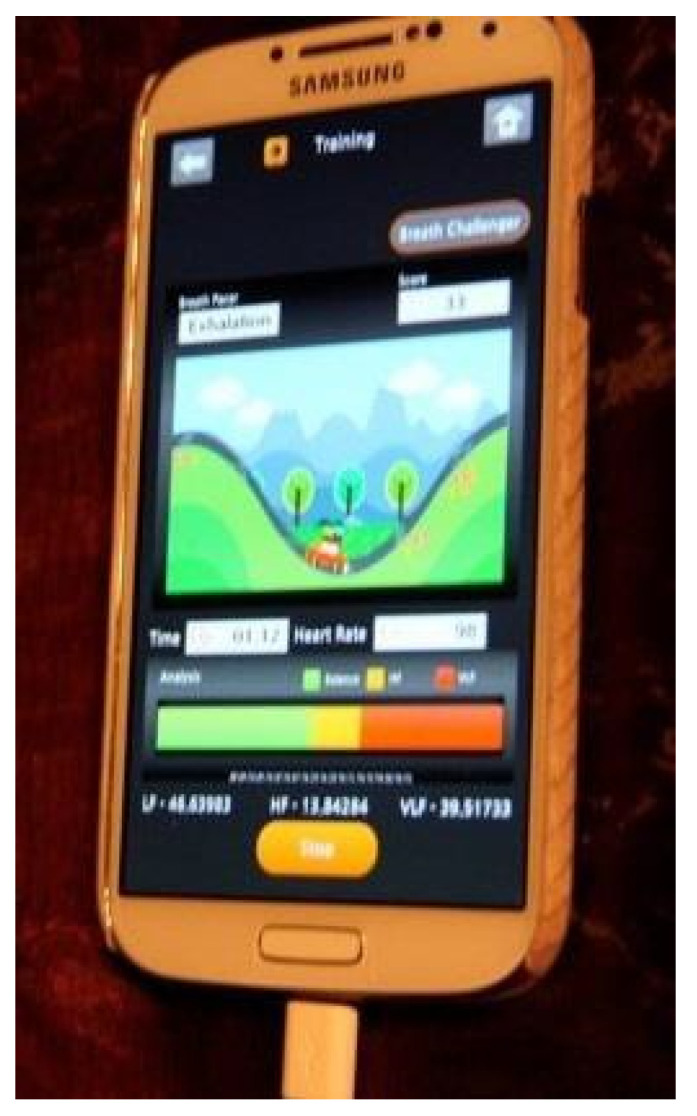
ResCalm.

**Figure 6:**
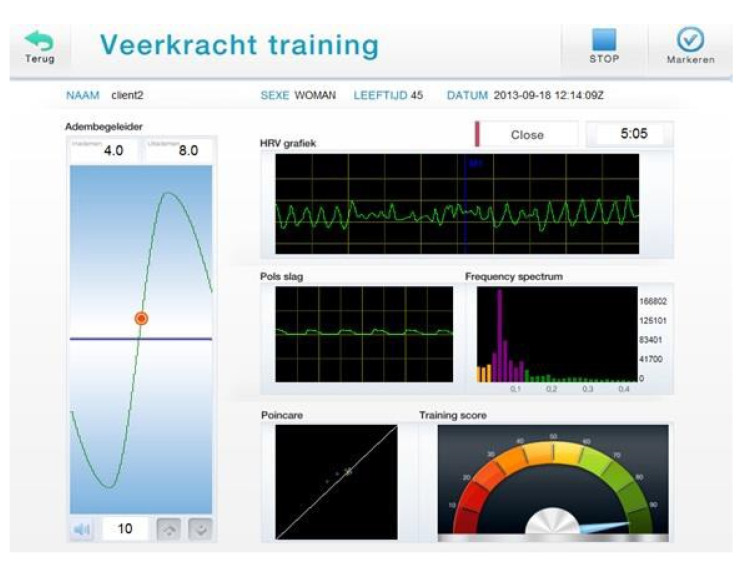
Balance Manager.

For Samsung phones the HRV biofeedback device ResCalm (Figure 5) has been developed in South Korea with 10 playful wave patterns like hills, mountains, and motivating wave movements. Data definition is the same as the Balance Manager (Figure 6) for Windows computer.

**Figure 7:**
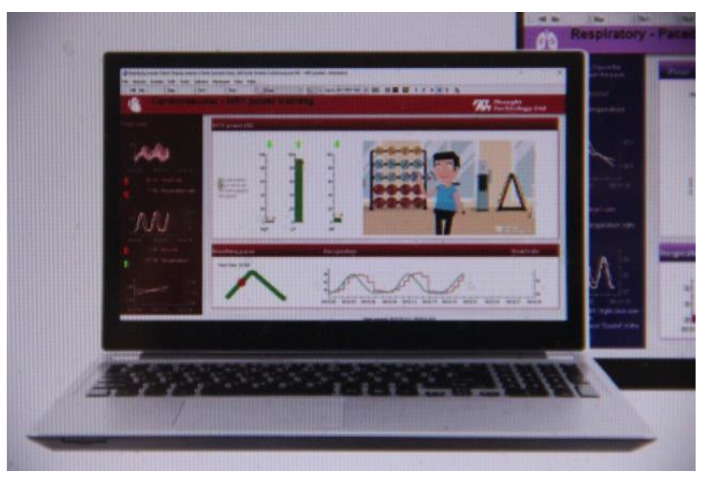
Infinity biofeedback.

Additionally, in the Infinity HRV biofeedback devices (Figure 7), playful pictures are effective in the treatment.

All these devices were very effective, because users can install their personal frequency and their preferred rate of inhalation–exhalation. While searching for different systematic reviews, we also found a study that used HRV-Biofeedback devices that only used the frequency of 0.1 Hz [63]. That study did not show significant results compared with the devices where a client can install a personal breathing frequency like Balance Manager, StressEraser Pro, and Infinity. 

In spite of the significance and efficacy of HRVB showed in this systematic review HRVB is not yet integrated into standard treatment. One of the most popular therapies is Acceptance and Commitment Therapy (ACT) [64]. In most trials ACT is reported to be superior or equally effective as cognitive behavioral therapy [65].Maybe it would be more efficient for our health system to integrate psychophysiology like ACT, HRV-Biofeedback and mindfulness in treatment of depression, PTSD, and anxiety disorders, because of the efficacy, more self-control of the client, and lowering the cost of treatment [66].

### Limitations

The search strategy was bias-free but limited to articles published in English.

## 5. Conclusions

More than 4000 studies that investigated HRV, show the relevance of HRV in neuroscience related to a range of medical and emotional disorders and especially stress related disorders. Stress related disorders have a connection to a disturbance of the Autonomic Nervous System and a dysregulated Vagus nerve.

This systematic review shows significant improvement of the non-invasive HRVB training in stress related disorders like PTSD, depression and panic disorder, in particular when combined with cognitive behavioral therapy or other TAU.

After critical appraisal from the 881 studies about depression, PTSD and anxiety, eight RCT studies and two related studies have been selected. The RCTs with control groups treatment as usual combined with muscle relaxation training, and a “placebo“-biofeedback instrument revealed significant clinical efficacy and better results compared with control conditions, mostly significant (*p <* 0.001).

In the depression studies average reduction at the Beck Depression Inventory scale was 64% (HRVB plus TAU) versus 25% (control group with TAU) and 30% reduction (HRVB) at the PSQ scale versus 7% (control group with TAU).

In the PTSD studies average reduction at the BDI scale was 53% (HRVB plus TAU) versus 24% (control group with TAU) and 22% (HRVB) versus 10% (TAU) with the PCL scale. Even with studies with groups from 26 to 60 participants there is significance efficacy, so the effect size is very interesting.

In the different meta-analyses, significant effects have been shown of HRVB in treatment of asthma, angina pectoris, coronary artery disease, sleeping disorders, prevention of postpartum depression, and stress and anxiety.

More research on the integration of HRVB in the treatment of stress related disorders in psychiatry is warranted. In addition, research focused on the neurophysiological mechanisms will solidify the scientific basis of HRVB.

Nevertheless, because financial support for behavioral research has not reached the level necessary to test thousands of, meta-analysis may be the best alternative for evaluating these effects.

## Figures and Tables

**Table 1:** The results of the selected studies.

RCT Studies HRVB for Treatment PTSD and Depression											
RELEVANCE							RESULTS				
Autor	Design	n	Domain	Setting	Period	Scale	Pre	Post	Reduction		Signif.
	RCT/SGT						Exp	Exp	Exp	Cont	Pre-Post
Zucker (2009)[28]	RCT HRVB vs. PMR	38	PTSD	1^e^ line	4 weeks daily	PCL	52.6	38.6	27%	18%	*p* < 0.05
						BDI-II	26.4	12.3	53%	24%	*p* < 0.05
Tan (2011) [29]	RCT HRVB vs. TAU	20	PTSD	Veteran hosp	8 weeks daily	PCL-S	64.8	54.4	16%	2%	*p* < 0.05
						CAPS	86.4	71.2	18%	9%	*p <* 0.001
Rene (2008) [30]	RCT HRVB vs. PMR	46	Depression	1^e^ line	8 weeks daily	BDI-II	35.0	7.8	78%	44%	*p <* 0.005
Chaudhri (2008) [31]	RCT HRVB+DBT vs sertraline	60	Depression	univ.hos	12 weeks daily	BDI-II	31.0	7.5	76%	29%	*p <* 0.001
						DERS	123.1	64.2	48%	8%	*p <* 0.001
Patron (2013) [32]	RCT HRVB vs. TAU	26	Depression after infarct	univ.hos	2 weeks daily	CES-D	15.3	8.9	42%	1%	*p* = 0.02
Van der Zwan (2019) [33]	RCT HRVB vs. waitinglist	50	Depression Anxiety Stress	University pregnant Women	5 weeks daily	DASS PSQI	5.45 6.55	2. 84.7	49% 21%	35% 10%	*p* = 0.039 *p* = 0.063
Karavidas (2007) [34]	SGT	11	Depression	univ.hos	10 weeks	BDI	26.0	12.5	52%	-	*p <* 0.001
Siepman (2008) [35]	HRVB vs. healthy	38	Depression	univ.h vs. stud	4 weeks 3× a Week	BDI	21.5	5.5	74%	-	*p <* 0.05
Thode (2019) [36]	RCT HRVB vs. TAU	37	MDD	LatinoHealth center	4 weeks	PHQ-9 GAD-7	16.42 11.08	10.85 6.50	40% 41%	7% 9%	*p* <0.05 *p* < 0.05
Lin (2016)[37]	Case control study	9	MDD Depression	Heroin users	5weeks 1× a week	BDI-II BDI cogn	23 19	18.3 14.3	20% 25%	-	*p* > 0.05
Lin (2019) [38]	RCT HRVB vs. med.care	48	MDD	3 hospitals	6 weeks	BDI-II PSQI	24.25 12.42	15.04 8.92	38% 28%	1% 5%	*p* = 0.007 *p* = 0.012

RCT: Randomized controlled trial; SGT: Single Group Trial; PMR = progressive muscle relaxation; DBT = dialectical behavioral therapy; PPD: postpartum depression; univ.hos = university hospital; BDI: Beck Depression Inventory; PCL: PTSD Check-List; CAPS: Clinical Administered PTSD Scale; CES-D: Centre Epidemiological Study-Depression; DERS: Difficulty in Emotion Regulation Scale; STAI: State-Trait Anxiety Inventory; PHQ-9: Patient Health Questionnaire; GAD-7: Generalized Anxiety Disorder.

**Table 2:** Search HRVB RCT studies.

Asthma [47]	*n* = 64	*p <* 0.003	USA
Angina Pectoris [48]	*n* = 63	*p <* 0.001	Canada
Angina Pectoris [11]	*n* = 154	sig	Taiwan
Anxiety [49]	*n* = 15	sig	South Korea
Anxiety [50]	*n* = 40	*p <* 0.05	USA
Cancer [51]	*n* = 5	*p <* 0.06	Belgium
Chronic fatigue syndrome [52]	*n* = 28	sig	Germany
Chronic Pain [53]	*n* = 20	*p <* 0.001	USA
Coronary artery disease [54]	*n* = 63	*p <* 0.001	USA
Coronary artery disease [55]	*n* = 210	*p* = 0.001	Taiwan
Depression (see Table 1)	*n* = 230	sig	USA, Italy, Taiwan, Netherlands
Emotion regulation [56]	*n* = 58	sig	Australia
Sleep apnea [57]	*n* = 853	sig	Brazil
Sleep [58]	*n* = 69	*p* = 0.001	Japan
Selfcontrol Psychotic sympt [59]	*n* = 84	*p* = 0.006	Germany
Stress and anxiety [18]	*n* = 484	Hedges g = 0.81	USA
Stressreduction [60]	*n* = 23	sig	Netherlands
Pediatric Irritable Bowel Syndr [61]	*n* = 24	sig	USA
Postpartum depression [62]	*n* = 55	*p <* 0.001	Japan
PTSD (see Table 1)	*n* = 97	*p <* 0.05	USA
Trait Anxiety [49]	*n* = 15	sig	South Korea
